# Industry-academia interface: building a spin-out

**DOI:** 10.1038/s44172-024-00207-2

**Published:** 2024-05-07

## Abstract

Daniel Perez Lopez is Co-founder and Chief Technology Officer at iPronics, a company dedicated to the development and commercialization of integrated programmable photonic circuits. His company focuses both on hardware advances for novel circuit and component architectures as well as software advances leading to the creation of fault-tolerant automated routines enabling advanced optical networking and processing, specially for AI infrastructure and intra-datacenter communications. As a young entrepreneur, Daniel shares with us his experiences and insights of the academia-industry transition and building a spin-out company from his university research.

**Figure:**
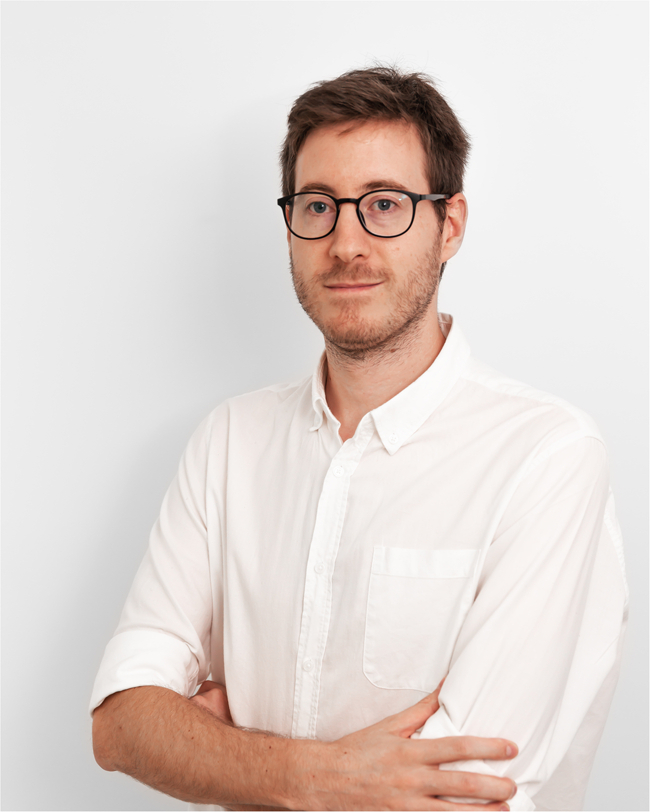
Photograph by Daniel Pérez López

1. Tell us about your career path to date.

My professional trajectory began after obtaining a bachelor’s degree in Telecommunications, propelling me into research. I initially focused on integrating radio-frequency and optical signals via on-chip integrated optics. My pursuits evolved through an M.Sc. in Tech, systems, and networks, culminating in a Ph.D. role at the Photonics Research Labs, Polytechnic University of Valencia.

My research transitioned towards general-purpose integrated programmable photonics. Delving into foundational components, circuits, and algorithms, I orchestrated work on a silicon photonic platform development. It was further supported by 4 months spent at the Optoelectronics Research Centre in the UK, where I could focus on the design and fabrication of the first silicon-based prototype. During my postdoctoral tenure, I got into teaching and mentored a group of 10 students who worked on programmable photonics projects.

Driven by my thesis results and the mutual conviction with my thesis supervisor, we founded iPronics in December 2019. I had to balance academic commitments with company endeavors, including a 1-year contract with a Canadian photonic company—Xanadu Quantum Technologies. After that, I transitioned to full-time CTO in 2021, channeling my expertise to drive iPronics’ innovation and growth.

2. Your product is based on the research which you did during your PhD. Tell us a little bit about it. How did you decide that this was a strong commercializable concept? As an individual with an eye on entrepreneurship, were there challenges you faced when dealing with intellectual property coming out of your research?

Commercialization revolves around addressing a problem with a viable solution. In our case, we pinpointed two distinct issues that shared a common remedy. First, after dedicating five years to integrated optics, we encountered three primary barriers hindering the development of a photonic chip/system: high costs (ranging from 100k€ to 1000k€), extensive timelines (spanning from 12 to 24 months), and knowledge gaps (involving risks, CAD tools, PDKs (define?), foundry variations, etc.).

Second, the escalating demand for flexibility and reconfigurability in optical processing and networking applications in AI infrastructure and intradatacenter communications became apparent. The resolution for both challenges lay in designing a software-defined optical chip that could be programmed post-fabrication, akin to the solutions pioneered by the electronics industry in the 1970s. The moment we achieved functional simulations and conducted initial experiments, the thrilling prospects of a promising commercial venture became glaringly evident.

Regarding intellectual property, when our company emerged from an academic research group, the university provided robust backing throughout the IP negotiation process. Nonetheless, it is worth noting that IP protection tends to be undervalued by Ph.D. students and some supervisors. A significant segment of the academic sphere still gauges success primarily through metrics like the h-index, thereby not always evaluating the patent versus journal dissemination decision with a composed mindset.

3. What were the key steps you took to move your ideas out of the lab and form the basis for a spin-out company? Highlight any challenges you faced during that transition.

In my academic journey, I laid useful groundwork for building programmable photonic systems, spanning from fundamental components and circuits to the intricate software layer. However, it became evident that harnessing the power of a product-oriented team was essential to amplify not just the quality and performance but also the speed of development.

I navigated through three pivotal stages to achieve this:

1. *Entrepreneurial training*: Recognizing the scarcity of entrepreneurship education in academia, I proactively sought out additional training. While my academic curriculum offered only limited exposure—two subjects at the Bachelor’s level and one during my Ph.D.—I took it upon myself to delve deeper. I dedicated a year to immerse myself in a photonic computing company in Canada and collaborated with seasoned business experts to grasp the distinctive nuances of steering a company, distinct from managing a research endeavor.
2. *Institutional support*: Research centers and universities and expert entrepeneours proved invaluable in providing foundational support to craft a solid business plan. Leveraging these resources became instrumental in setting the stage for a viable business trajectory.
3. *Legal guidance*: Transitioning from a laboratory setting to the dynamic landscape of a startup, I encountered a maze of complex legal intricacies—IP negotiation, shareholder agreements, and initial employee contracts. Recognizing the necessity, we sought robust legal support. Partnering with specialized law firms, such as RCD in my case, proved pivotal in securing tailored and expert guidance essential for the startup’s foundation.

4. You now have experience both in academia and in running a company. What are the similarities and differences between academic life and managing a spin-out?

The common threads between academic research and entrepreneurial ventures are striking: they both demand a multidisciplinary approach, prioritize excellence as a defining factor, and necessitate an unceasing pursuit of knowledge.

In academia, researchers often embody the versatility of Swiss army knives, extending beyond pure research to encompass administrative responsibilities, team building, funding pursuits, and more. Similarly, the entrepreneurial researcher must navigate this multifaceted landscape, simultaneously developing innovative solutions while orchestrating organizational coherence and decisively prioritizing the next strategic steps. Furthermore, the relentless pursuit of excellence stands as a cornerstone in both realms. The ethos of unwavering effort is intrinsic to achieving ambitious goals, fostering an environment where excellence becomes the modus operandi. A slight difference might be that in in business, you need to learn when a feature is good enough for a specific application and focus on the key open challenges.

However, notable disparities surface when considering risks, team dynamics, and the typical timescales. Research projects typically aim at validation or the discovery of limitations. Conversely, a company’s initiatives are rooted in assumptions about reaching specific market milestones. Delays and misjudgments in project development or market analysis can profoundly impact the company’s trajectory. Moreover, the team dynamics diverge significantly. Academic research often involves smaller, focused teams—often limited to a handful of individuals—constraining the scope of multidisciplinary collaborations within the institution. Conversely, entrepreneurial endeavors thrive on collaborative, multidisciplinary efforts within larger teams, fostering expansive and transversal innovations. Finally, the tempo and pressure appear more pronounced within the entrepreneurial landscape, although I acknowledge that the perception of pressure is subjective and varies across contexts.

Ultimately, the crux lies in adept organizational skills and the ability to prioritize tasks in both domains. Personally, I’ve found immense excitement in the continual learning curve presented by the team—each individual’s insights, emotions, and passion serve as catalysts that, when harnessed effectively, significantly elevate the company’s performance.

5. Tell us more about your life now. What do you spend your time doing? What do you love and what do you not like so much about your role?

My role as CTO and cofounder involves day-to-day technical tasks while also having a strategic vision for the company’s long-term growth. My role requires a balance between technical expertise, leadership skills, and business acumen. Precisely, I steer our tech strategy to sync with our business objectives. My role involves leading and nurturing our tech team, fostering a vibrant culture, and sourcing and growing talent. I oversee product development, ensuring quality and relevance while scouting for new tech and market needs to innovate through meetings with customers. In addition, I manage project budgets, allocating resources for maximum impact. On the financial peace, I support fundraising processes and help the business team to cultivate valuable partnerships.

6. It must be a challenging task to probe the potential market when you are offering a one-of-a-kind product. What do you think about growth and the future of your product and your company? What are the best practices to collect the necessary data and meet with the right people?

Navigating a unique product’s market potential is indeed a challenge. For us, growth lies in a two-fold approach: educating the market about our innovation and adapting based on their response. We prioritize a mix of direct market engagement, gathering insights from early adopters, and employing data analytics to understand user behavior. Leveraging industry connections, partnerships, and targeted events aids in meeting the right stakeholders. Best practice involves a blend of quantitative and qualitative data collection, employing meetings, user testing, and interviews. Continuous refinement and agile adaptation based on user feedback remain pivotal in our growth strategy, but you always need to prioritize the points to evolve and avoid distractions. As we pioneer the niche of software-defined photonic circuits, our future hinges on the symbiotic evolution of our product with the market’s needs, all while maintaining a keen eye on technological advancements.

7. What do you think of the evolving global market of spin-out companies?

The global market for spin-out companies is expanding, fueled by a surge in technological advancements, entrepreneurial spirit, and support from universities, governments and investors, particularly in AI-related domains. In the particular area of integrated optics, spin-outs often possess niche expertise but face challenges in establishing a good product-market fit and the associated traction due to their pioneering nature. Distinguishing features include their innovative core, stemming from research or breakthrough technologies, yet they have a daily battle with scalability and commercialization hurdles.

A key challenge is sourcing a skilled workforce proficient in both deep tech and business domains. There’s a demand for talent adept at translating complex innovations into viable products. Bridging this skill gap remains critical for sustained growth. Collaborations between academia, industry, and government can foster a conducive environment for spin-outs, providing funding, mentorship, and resources to connect with the market at the earliest stages. Being late on market engagement could result in losing against the competition or in developing an incredible technology with no practical users.

8. Finally, imagine someone at the beginning of their spinout journey. What top three pieces of advice would you give, based on your experience and lessons learned?

First, selecting the initial team and co-founders is crucial. The collective should bring diverse skills and expertise to ensure rapid progress from the start. This might involve outsourcing for specialized tasks like legal support or specific market analysis. It is vital to choose individuals you trust deeply and who share the same level of commitment. Equally important, get direct expertise from a short or medium-term stay in a startup or medium-sized company. Strive to understand what succeeds and what doesn’t.

Second, placing the customer’s problem and finding the right product-market fit at the core of all decisions is essential. Time is your most valuable resource, and using it effectively sets you apart, especially when competing against larger companies or well-funded spin-offs. Focusing on solving the right problems and avoiding distractions is a key differentiator.

Finally, maintaining a balance between data-driven product-market fit insights and your instinct and deep vision is crucial, especially in new deep-tech product development. There’s no manual or single expert who holds all the answers, so continuous monitoring and evaluation are vital to positioning your product in the market effectively.

As an additional point to the first one, periodically listening to your team is invaluable. Attending to their insights can broaden your perspective, boosting the team’s motivation and enhancing overall performance.

This interview was conducted by Anastasiia Vasylchenkova, Associate Editor, *Communications Engineering*, and Rosamund Daw, Chief Editor, *Communications Engineering*

